# Unveiling the Power of Platelet-to-Lymphocyte Ratio as a Game-Changer in Late-Onset Neonatal Sepsis Diagnosis

**DOI:** 10.3390/children12060687

**Published:** 2025-05-26

**Authors:** Dilek Kahvecioğlu, Melda Taş

**Affiliations:** Department of Pediatrics, Neonatalogy, Ankara Training and Research Hospital, University of Health Sciences, 06018 Ankara, Turkey; melda.tas@saglik.gov.tr

**Keywords:** PLR, late-onset neonatal sepsis, NLR

## Abstract

**Background/Objectives**: The present study evaluated the diagnostic utility of underutilized parameters derived from complete blood count (CBC) analysis in identifying late-onset neonatal sepsis (LOS). The parameters evaluated included the nucleated red blood cell count (NRBC), neutrophil-to-lymphocyte ratio (NLR), red cell distribution width (RDW), plateletcrit (PCT), and platelet-to-lymphocyte ratio (PLR). **Methods:** This was a retrospective, single-center, case-control study in a tertiary neonatal intensive care unit. The study included 38 neonates diagnosed with LOS, and 22 healthy control subjects. The data collected encompassed demographic characteristics, clinical findings, and laboratory values, including complete blood count (CBC)-derived parameters, C-reactive protein (CRP) levels, and blood cultures. Statistical analyses were performed to assess differences between groups and the diagnostic performance of key parameters via receiver operating characteristic (ROC) curves. **Results:** The results of the study are as follows: A set of notable discrepancies were identified in a number of parameters when comparing the LOS and control groups. Elevated levels of C-reactive protein (CRP), platelet count, platelet-to-lymphocyte ratio (PLR), lymphocyte percentage, and neutrophil-to-lymphocyte ratio (NLR) were found to be associated with LOS. Concurrently, decreased hemoglobin, hematocrit, neutrophil percentage, NRBC percentage, and NLR were also associated with LOS. PLR exhibited the most robust diagnostic efficacy, with a cutoff value of 45.24 attaining 81.6% sensitivity, 61.9% specificity, and an area under the curve (AUC) of 0.787 (95% CI: 0.671–0.903). The application of a logistic regression analysis indicated that the PLR emerged as the most salient independent predictor of LOS (odds ratio [OR]: 1.071; 95% confidence interval [CI]: 1.009–1.135; *p* = 0.023). **Conclusions:** CBC-derived parameters, particularly the PLR, have been shown to offer promising diagnostic value for LOS. These findings support the incorporation of these accessible and cost-effective biomarkers into clinical practice for the early diagnosis and management of LOS, warranting further validation in larger, multicenter studies.

## 1. Introduction

Despite the advances in neonatal medicine, neonatal sepsis remains a significant cause of morbidity and mortality. The incidence of neonatal sepsis varies widely, ranging from 1 to 4 cases per 1000 live births in high-income countries to as high as 49 to 170 cases per 1000 live births in low- and middle-income countries, where the case fatality rate can reach up to 24% [1,2,3]. Survivors of neonatal sepsis, whether culture-positive or culture-negative and treated with antibiotics, face an increased risk of adverse neurodevelopmental outcomes, including cerebral palsy, hearing loss, visual impairment, and cognitive delays.

Early recognition and prompt treatment are critical for improving outcomes in neonatal sepsis. However, arriving at an accurate diagnosis is often challenging due to the subtle and non-specific nature of the clinical signs exhibited. Laboratory tests, including blood cultures, are imperative for identifying the causative agents and directing appropriate antibiotic therapy. However, factors such as prior antibiotic exposure can reduce the sensitivity of these tests, potentially leading to underdiagnosis. Therefore, a multitude of biomarkers, including but not limited to IL-6, IL-2, IL-4, TNFα, presepsin, proadrenomedullin, sTREM-1, and serum amyloid A, have been utilized in the diagnostic process of sepsis [3,4,5,6].

Despite the recent development of new diagnostic tests, access to these tests remains limited and expensive, especially in low- and middle-income countries. A complete blood count (CBC) is a widely used, easily accessible, and cost-effective test with proven validity in numerous studies [7,8]. Although the popularity of CBC has diminished with the advent of newer tests, we posit that the use of newly available parameters within the CBC will enhance its specificity and sensitivity in the diagnosis of sepsis. It is evident that these underutilized parameters offer profound insights into immune responses and inflammatory processes. Consequently, they possess the potential to play a crucial role in the early diagnosis and management of sepsis. Therefore, CBC is likely to retain its value as a critical diagnostic tool alongside emerging technology.

The neutrophil-to-lymphocyte ratio reflects the balance between innate and adaptive immune responses. Studies have shown that NLR is significantly altered in septic neonates. A 2023 meta-analysis revealed a pooled sensitivity of 79% and specificity of 91% for NLR in diagnosing neonatal sepsis, indicating its potential as an inflammatory marker [9].

The platelet-to-lymphocyte ratio has been widely studied in adults as a marker of systemic inflammation and has recently been explored in neonatal populations. During sepsis, platelet activation and lymphopenia can cause PLR to increase, reflecting the severity of the inflammatory response [10]. Although data on its use in late-onset neonatal sepsis (LOS) are limited, emerging evidence supports its diagnostic potential.

The red cell distribution width is a measure of the variability in red blood cell size and is frequently elevated in cases of systemic inflammation. In neonates, elevated RDW has been associated with the presence of sepsis and unfavorable clinical outcomes. A meta-analysis demonstrated that RDW can effectively differentiate between septic and non-septic neonates, exhibiting a high degree of diagnostic accuracy, with sensitivity and specificity levels of 88% and 90%, respectively [11].

Plateletcrit represents the total platelet mass and may increase in response to thrombocytosis during inflammation. Although less studied than other indices, it may offer additional value in sepsis diagnostics when combined with other CBC parameters (8).

Nucleated red blood cells are typically absent in healthy term neonates but can appear in peripheral blood in response to hypoxia or systemic stress, including sepsis. Elevated NRBC counts may thus serve as a supportive indicator of disease severity or systemic inflammation [12].

Given the accessibility and affordability of CBC testing, the investigation of these derived parameters could enhance diagnostic precision for neonatal sepsis, particularly in resource-limited settings. This study aims to assess the diagnostic utility of NLR, PLR, RDW, PCT, and NRBC in identifying cases of late-onset neonatal sepsis.

## 2. Materials and Methods

The study was conducted in the University of Health Sciences Ankara Education and Training Hospital, a tertiary neonatal intensive care unit with 1500 deliveries per year and 450 NICU admissions, between September 2023 and August 2024. The study was designed as a single-center, retrospective, case-control study. Data were collected for 38 patient and 22 control groups. The study received ethical approval from the Ankara Health Sciences University Ethics Committee for Clinical Trials on 7 December 2023 (approval number: 1482/2023) before initiation.

### 2.1. Selection of Groups

#### 2.1.1. Patient Group

Patients diagnosed with late-onset neonatal sepsis within the first 3–30 days of life and followed up in the NICU were included in the study retrospectively. A form evaluating demographic characteristics and hospitalization features was filled out. In this form, the gestational age, mode of delivery, birth weight, gender, APGAR score, clinical findings, maternal age, and any accompanying maternal illnesses were investigated in both the patient and control groups of infants. Also, the complete blood count and CRP levels of the patient and control groups were determined.

Patients were included in the study if they were hospitalized in the NICU with a preliminary diagnosis of LOS and if antibiotics were initiated based on the neonatologist’s suspicion of sepsis using the Pediatric Committee of the European Medicines Agency [EMA] scores. A detailed physical examination was performed on all patients. Infants were grouped as having LOS if they showed at least two clinical and two laboratory signs that matched the EMA neonatal sepsis standards. These signs included irritability; lethargy; hypotonia; body temperature; cardiovascular instability; skin and subcutaneous lesions; respiratory instability; gastrointestinal symptoms; and laboratory findings such as a leukocyte count of <20,000/mm^3^ or >40,000/mm^3^, an immature/total neutrophil ratio of ≥0.2, a platelet count of <100,000/mm^3^, a C-reactive protein (CRP) level of >1.5 mg/dL. >20,000/mm^3^ or <4000/mm^3^, an immature/total neutrophil ratio: ≥0.2, a platelet count: <100,000/mm^3^, C-reactive protein: >1.5 mg/dL, hyperglycemia (>180 mg/dL), hypoglycemia (40 mg/dL), and metabolic acidosis (BE > 10 mEq/L, lactate > 2 mmol/L) [13]. Blood analyses were obtained from patients in the patient group at 10 days (min-max: 4–30 days).

Patients were excluded from the study if they had been diagnosed with early-onset neonatal sepsis, transient tachypnea of the newborn, congenital pneumonia, congenital heart disease, hypoxia, intraventricular hemorrhage, metabolic disorders, congenital hematologic abnormalities, and immunologic disorders or neonatal abstinence syndrome.

#### 2.1.2. Control Group

A total of 22 infants, including both term and preterm births (with gestational ages ranging from 32 to 42 weeks), were included in the study. These infants were born to healthy mothers without any underlying infectious diseases. The mothers of these infants were subsequently monitored during their pregnancies by the obstetrics and gynecology service. The control group comprised infants born to healthy mothers who did not have any infectious diseases, premature prelabor ruptures of the membranes, or proven or suspected infectious diseases. Additionally, these infants did not exhibit any clinical signs of sepsis and were not receiving antibiotic treatment. A cohort of patients diagnosed with perinatal asphyxia, transient tachypnea of the newborn, respiratory distress syndrome, pulmonary hypoplasia, congenital diaphragmatic hernia, congenital and acquired heart diseases, adrenal diseases, intraventricular hemorrhage, hypoxic-ischemic encephalopathy, congenital metabolic diseases, congenital blood, immunologic disorders, or other conditions that could affect complete blood count (CBC) parameters were excluded from the control group. Blood samples were collected from the patients in the control group at the 24-h mark after birth.

### 2.2. Laboratory Methods

#### 2.2.1. Collection and Storage of Blood Samples

Patients admitted to the NICU, including both term and preterm infants (38 cases with LOS and 22 in the control group), were enrolled in the study. When clinical sepsis was suspected in patients, a comprehensive analysis was conducted, including complete blood count (WBC, PLT, NRBC, NLR, RDW, platecrit (PCT), PLR, neutrophil and lymphocyte percentages, CRP, and blood culture).

Samples for complete blood count, CRP, and blood culture (and other cultures if clinically indicated) were collected immediately after the suspicion of clinical sepsis. These samples were then processed in the Medical Biochemistry and Medical Microbiology laboratories.

#### 2.2.2. Laboratory Investigations

Complete blood count (CBC): Approximately 1 mL of blood was collected in a hemogram tube containing K2 EDTA to determine the white blood cell count, platelet count, and percentage of immature granulocytes. CBC parameters were measured using the Sysmex XN-2000 (Norderstedt, Germany) and Sysmex XN-3000 (Norderstedt, Germany) analyzers with the fluorescent flow cytometry method.

CRP: For the detection of acute phase reactants, approximately 0.5–1 mL of blood was collected in a dry tube, followed by centrifugation at 5000 rpm for 10 min in the laboratory. The supernatant (serum) portion was separated. Quantitative measurements of CRP levels were performed using an appropriate kit and the immunoturbidimetric method on the Roche Cobas 6000 c501 analyzer (Roche Diagnostic, Indianapolis, IN, USA).

Blood Culture: Blood cultures were obtained via peripheral venipuncture (1–2 mL) and inoculated into BD BACTEC Peds Plus bottles. Cultures were incubated in a BACTEC FX 200 system (Becton Dickinson, Sparks, MD, USA).

### 2.3. Statistical Analysis

Descriptive statistics including mean, standard deviation, median, minimum, and maximum values for continuous variables, and number and percentage for discrete variables, were presented. The normality of the data was assessed using the Shapiro–Wilk test. The student’s *t*-test was used for continuous variables with normal distribution, and the Mann–Whitney U test was used for variables without normal distribution to compare differences between patient and control groups. For a comparison of nominal variables (in cross-tables), Chi-square and Fisher’s Exact tests were utilized. PLR, NLO, CRP, and WBC were included in the multivariant model to detect the independent factors of LOS. The diagnostic performance of PLR values was evaluated using the area under the ROC curve (AUC). The optimal cutoff point was calculated using the Youden’s Index. IBM SPSS version 20 (SPSS Inc. Chicago, IL, USA) was used for analysis, and a *p*-value <0.05 was considered statistically significant.

## 3. Results

Our study included 38 patients diagnosed with LOS who were followed up in the neonatal intensive care unit, along with 22 control subjects followed up with their mothers in the obstetrics and gynecology service. The demographic, clinical, and laboratory data of the patients and the control group were examined.

The demographic characteristics and laboratory findings of the infants in the patient and control groups are provided in Table 1.

PLR, NLO, CRP, and WBC were included in the multivariant analysis, and PLR (OR: 1.071, CI: 1.009–1.135, and *p*: 0.023) was the most parameter independently associated with LOS.

**Figure 1:**
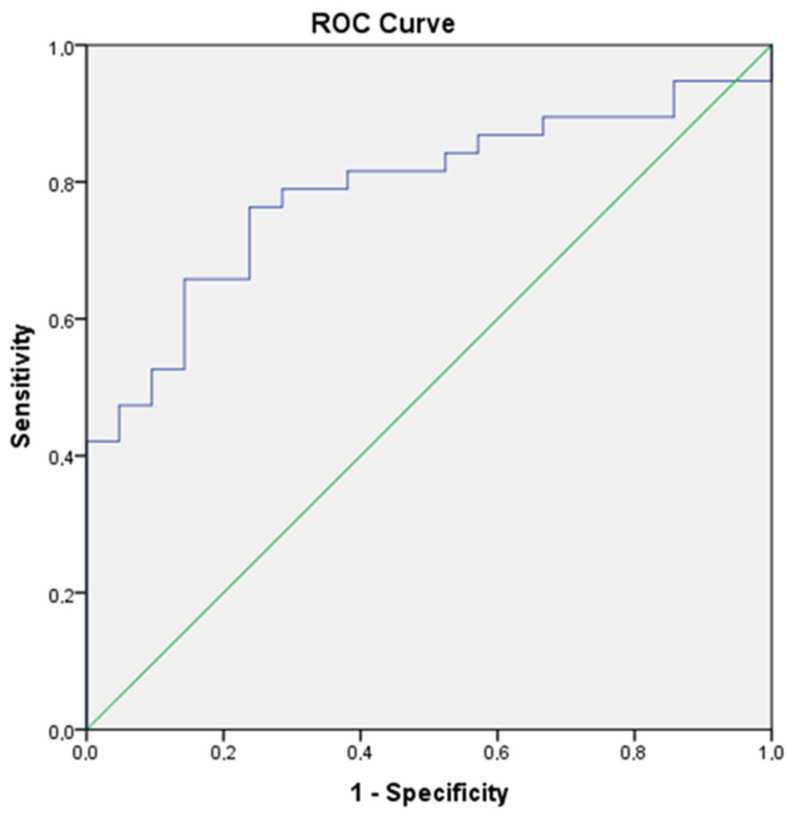
Roc curve of PLR (AUC of 0.787 (95% CI: 0.671–0.903). The optimal cutoff value was 45.24, with a sensitivity of 81.6%, specificity of 61.9%, PPV of 79.5%, and NPV of 65%, *p* < 0.001).

ROC curve analysis showed an area under the curve (AUC) of 0.787 (%95CI: 0.671–0.903), a cut off score of 45.24, a sensitivity of 81.6%, and a specificity of 61.9% and PPV %79.5 (%95CI: 65.2–90.1) *p* < 0.001, NPV %65 (%95 CI: 43.2–83.2) *p* < 0.001 (Figure 1).

In 23% of patients (*n* = 9), positive blood cultures were identified. Upon analyzing the culture results, *S. epidermidis* was found in 5% (*n* = 2) of cases, *S. haemolitycus* in 7% (*n* = 3), ESBL(+) Klebsiella in 5% (*n* = 2), and *E. coli* in 2% (*n* = 1). No growth in blood cultures was observed in 77% of the patients in the LOS group and all of the control patients. A subgroup analysis was performed on the culture-positive patient group. The results showed that the gestational week, birth weight, and APGAR scores of the culture-positive patients were lower than those of the culture-negative group (*p* < 0.05). However, no significant differences were detected in terms of laboratory parameters (Table 2).

## 4. Discussion

In our study, we aimed to explore the diagnostic utility of underutilized parameters derived from CBC analysis in the diagnosis of late-onset neonatal sepsis. Our findings suggest that elevated levels of CRP, platelet count, PCT, lymphocyte percentage, and PLR, combined with decreased levels of Hb, Hct, neutrophil percentage, NRBC percentage, and NLR, are strongly associated with LOS. These findings highlight the potential of CBC-derived parameters, particularly PLR, as valuable diagnostic markers for LOS in neonates.

The gold standard method for confirming sepsis diagnosis is the detection of the pathogen in blood cultures; however, it takes 48–72 h to obtain the results. Factors such as proper blood collection techniques, swift and appropriate processing, adherence to sterile protocols, and execution by an experienced person affect the positivity rate of blood cultures. Unfortunately, even in the presence of serious infections, pathogens often cannot be identified in blood cultures [14]. In a study by Tallur and colleagues on neonatal sepsis, the culture positivity rate was found to be 64.87%. Despite being a confirmatory test for sepsis, blood cultures are negative in many neonates showing clinical features of sepsis [15]. In our study, 23% (*n* = 9) of patient infants had positive blood cultures. No significant differences were detected in terms of laboratory parameters in the culture (+) positive group. Due to the limited number of positive cultures, a comparison between the pathogens and predisposing factors of the patients could not be made. Therefore, our study is limited in this regard.

CRP is a widely used acute-phase reactant in the diagnosis of neonatal sepsis due to its easy accessibility, low cost, and rapid results [16]. It begins to be released approximately 4–6 h after inflammation and peaks at 24–48 h. CRP levels decrease proportionally with the reduction in inflammation [17,18]. Since CRP positivity can also be observed in non-septic events (trauma, asphyxia, RDS, IVH, and MAS), its combined use with other biomarkers aiding in diagnosis is recommended. In a study by Forrest and colleagues, serially measured CRP was found to be more beneficial and guided the termination of existing [18]. In our study, the median serum CRP level of patient infants was 7.15 mg/L (range: 0.3–229.6), which was significantly higher than the infants in the control group. Our study, along with other similar studies, supports this practice, but our study does not include repeated measurements, which limits our ability to address this issue.

During neonatal sepsis, various changes occur in neutrophils and lymphocytes. The study conducted by Hornik et al. showed that low WBC counts, low ANC, and high I: T neutrophil ratios were associated with increased odds of infection (highest odds ratios: 5.38, 6.84, and 7.97, respectively) and thus concluded that all these markers have high specificity and NPV and low sensitivity [19]. In our study, patient infants had lower neutrophil percentages and higher lymphocyte percentages compared to the control group, which were statistically significant. These findings indicate that these laboratory parameters can be diagnostically utilized in the diagnosis of LOS.

The neutrophil-to-lymphocyte ratio is considered to be a more sensitive indicator for microbial infection. It rises rapidly after being infected and is often associated with disease severity [20]. There have been increasing studies demonstrating that NLR would be of clinical significance for the diagnosis of neonatal sepsis and would be associated with the severity and prognosis of the disease [21]. In our study, the median NLR value of patient infants was 0.9 (range: 0.09–11), which was significantly lower compared to the control group. In LOS, NLR is expected to decrease in the diagnosis of LOS because of lymphocyte dominance. This suggests its potential use in diagnosing LOS.

Pro-inflammatory cytokines associated with sepsis have been shown to increase RDW by suppressing erythrocyte maturation and reducing their half-life. A prospective observational study compared the RDW values of septic neonates with those of the control group. RDW levels were significantly higher among neonatal sepsis cases (19.9%) than among controls (18.9%), with a *p*-value of <0.001. RDW levels were significantly higher among nonsurvivors than survivors (*p* < 0.003) [22]. In our study, we could not find a significant difference between groups according to RDW levels.

Plateletcrit represents the total platelet mass and may increase in response to thrombocytosis during inflammation. Plateletcrit may vary with inflammation and increased platelet consumption/production. Although less studied than other indices, higher PCT levels are associated with neonatal sepsis and respiratory distress in premature infants [23]. In the present study, elevated PCT values were observed in the LOS group in comparison with the control group. This finding suggests that this parameter, which has not been previously studied in the context of LOS, may warrant consideration as a significant diagnostic tool for sepsis.

Nucleated red blood cells are a type of erythrocyte precursor that is present in the bone marrow of humans of all ages as part of the process of erythropoiesis. These cells are seldom detected in the circulatory systems of healthy adults; however, they have been identified as present in the circulatory systems of fetuses and neonates. An NRBC count is a cost-effective laboratory test that is currently rarely used in everyday clinical practice. It is most often used in the diagnosis of hematological diseases or disorders relating to erythropoiesis, anemia, or hemolysis. Nevertheless, as indicated by the findings of numerous studies, its potential exists for utilization as a biomarker in the diagnostic process of sepsis [24]. In the present study, we observed that NRBC levels were lower in the study groups compared to the control groups. One potential explanation for this finding is the time difference in blood collection between the groups. It is possible that the decrease in NRBC levels after birth may have influenced our results.

Studies reported that platelet and lymphocytes play a critical role in inflammation. The inflammatory status resulted in accelerated megakaryocyte proliferation and associated thrombocytosis. Increased platelet counts and decreased lymphocyte counts have been showed to be related to both aggregation and inflammation. On the other hand, a microbial infection can cause an increase of total leukocyte and neutrophil counts. The platelet to lymphocyte ratio is a newly and easily calculated value, which was shown upon diagnosis of inflammatory diseases in adults. PLR is increased in the inflammatory response as the microcirculation of the body is altered, the permeability of blood vessels is increased, platelets are activated, and a large number of platelets are aggregated, which in turn aggravate the inflammatory response of the body [25]. PLR predictivity was shown in the diagnosis of EOS. But there is a lack of literature concerning its usage in the diagnosis of LOS [10,26,27]. In the meta-analysis conducted by Bai et al., they showed that data from 3 studies (SEN, SPE, and AUC of PLR) were 0.82, 0.82, and 0.87, respectively. But this meta-analysis includes both EOS and LOS patients [9]. In our study, there was a cut off score of 45.24 of PLR (a sensitivity of 81.6% and a specificity of 61.9%) with PPV of 79.5% and NPV of 65%. In the logistic regression analysis, PLR was found to be the most effective parameter associated with LOS (OR: 1.071, CI: 1.009–1.135, and *p*: 0.023). These findings support the use of PLR in the diagnosis of late neonatal sepsis.

The limitations of our study include its single-center design, which may limit the generalizability of the findings. Furthermore, we acknowledge that hematologic parameters in neonates undergo significant changes in the first few days of life [28]. In this study, blood samples for the sepsis group were collected at the time of clinical diagnosis, while control group samples were obtained within the first 24 h of life. This discrepancy represents a potential limitation, as early neonatal hematologic changes could affect CBC-derived parameters. We propose that future prospective studies should use strictly age-matched controls to eliminate this confounding factor. Although hematological changes in both term and preterm infants manifest differently in the postnatal period, this variation can be mitigated by ensuring that both groups have a comparable mean gestational age. In subsequent studies, it will be advisable to undertake a distinct evaluation of preterm and term groups to further investigate and potentially eliminate these observed discrepancies. Moreover, our study did not include repeated measurements to assess the dynamic changes in biomarkers during treatment, which could provide additional insights into the response to therapy.

## 5. Conclusions

In conclusion, although the clinical manifestations of neonatal sepsis are often non-specific, CBC-derived inflammatory markers—particularly CRP, PLR, and NLR—may contribute to improved diagnostic accuracy, especially in late-onset cases. Our findings highlight PLR as a promising and easily obtainable marker with potential clinical utility. Given that PLR is derived from routine complete blood counts, it represents a cost-effective and widely accessible diagnostic tool. These advantages make it a practical candidate for integration into neonatal sepsis screening protocols. Nevertheless, larger, multicenter studies are warranted to validate these findings and further define the role of PLR and related biomarkers in the early detection and management of neonatal sepsis.

## Figures and Tables

**Table 1:** Sociodemographic and laboratory findings of groups.

	LOS (*n* = 38)	Control (*n* = 21)	*p*
Gestational Age (week)	37.2 (28–41)	38 (33–41.6)	0.349
Birth Weight (gr)	2735.52 ± 799.78	2993.47 ± 542.71	0.148
Mother Age (year)	28.02 ± 6.82	28.47 ± 7.44	0.815
APGAR1	8 (3–9)	8 (6–10)	0.007
APGAR5	9 (4–10)	9 (8–10)	0.062
Birth Type NSVD C/S	23 (60.5%) 15 (39.5%)	9 (42.9%) 12 (57.1%)	0.192
Gender Girl Boy	23 (60.5%) 15 (39.5%)	9 (42.9%) 13 (57.1%)	0.192
Platelet	335,500 (59,000–779,000)	278,000 (237,000–339,000)	0.012
Hb (g/dL)	14.5 (±3.09)	19.5 (±2.17)	<0.001
HTC (%)	43.2 (±8.9)	54.8 (±5.7)	<0.001
WBC (10^3^/mm^3^)	12,225.7 (±6137.3)	20,027.1 (±5188.8)	<0.001
RDW (%)	16.2 (±1.7)	16.9 (±1.4)	0.139
Neutrophile%	39.2 (±19.0)	58.2 (±6.8)	<0.001
Lymphocyte%	43.25 (7.7–70.4)	27 (22.5–57.7)	0.010
% NRBC	0.1 (0–4.6)	0.7 (0.1–5.2)	<0.001
ANC (10^3^/mm^3^)	3525 (12.1–2800)	12,870 (2770–15,830)	<0.001
ALC (10^3^/mm^3^)	4720 (±1886)	5432.3 (±1383.6)	0.135
NRBC count	0.01 (0–15.2)	0.17 (0.02–0.52)	<0.001
MPV	10.4 (±1.2)	9.9 (±1)	0.146
PCT	0.36 (±0.15)	0.23 (±0.06)	<0.001
NLR	0.9 (0.09–11)	2.28 (0.5–3.3)	<0.001
PLR	70.31 (13.88–306.52)	48.98 (40.99–71.47)	<0.001
CRP (mg/L)	7.15 (0.3–229.6)	0.5 (0.2–2.7)	0.003

NSVD: normal spontaneous vaginal delivery; C/S: Cesarian section; Hb: hemoglobin; HTC: hematocrit; WBC: white blood cell; RDW: red cell distribution width; NRBC: nucleated red blood cell; ANC: absolute neutrophile count; ALC: absolute lymphocyte count; MPV: mean platelet volume; PCT: platecrit; CRP: C reactif protein; LOS: Late onset sepsis.

**Table 2:** Subgroup analysis of culture-positive patients.

	Culture (-) (*n* = 29)	Culture (+) (*n* = 9)	*p*
WBC (10^3^/mm^3^)	12,497 ± 6356	11,558 ± 5797	0.675
Platelet	352,000 (59,000–680,000)	317,000 (145,000–652,000)	0.748
Hb (g/dL)	14.53 ± 3.31	14.72 ± 2.61	0.866
HTC (%)	43.16 ± 9.74	43.46 ± 7.02	0.927
RDW (%)	16.03 ± 1.73	16.69 ± 1.64	0.291
Neutrophile%	37 ± 18.95	44.62 ± 19.21	0.270
Lymphocyte%	44.90 (9–65.7)	37.9 (7.70–55.5)	0.104
% NRBC	0.1 (0–4.6)	0.15 (0–2.6)	0.854
ANC (10^3^/mm^3^)	3140 (12.1–28,000)	3980 (1550–15,140)	0.430
ALC (10^3^/mm^3^)	5070 ± 1880	3860 ± 1680	0.073
NRBC count	0.01 (0–15.2)	0.15 (0–0.2)	0.665
MPV	10.17 ± 1.15	10.95 ±1.40	0.085
PCT	0.35 ± 0.16	0.39 ± 0.15	0.442
NLR	0.8 (0.09–9.43)	1.05 (0.4–11)	0.071
PLR	73.46 (13.88–154.7)	83.06 (42–306.5)	0.079
CRP	4.9 (0.3–44.1)	14.9 (0.3–229.3)	0.274

Hb: hemoglobin; HTC: hematocrit; NLR: neutrophil/lymphocyte ratio; PLR: platelet/lymphocyte ratio; WBC: white blood cell, RDW: red cell distribution width; NRBC: nucleated red blood cell; ANC: absolute neutrophile count; ALC: absolute lymphocyte count; MPV: mean platelet volume; PCT: platecrit; CRP: C reactif protein.

## Data Availability

The data presented in this study are available on request from the corresponding author due to in case of need.
